# Patient satisfaction with the quality of nursing care†

**DOI:** 10.1002/nop2.237

**Published:** 2019-01-04

**Authors:** Anita Karaca, Zehra Durna

**Affiliations:** ^1^ Florence Nightingale Hospital School of Nursing Istanbul Bilim University Istanbul Turkey

**Keywords:** nursing care, patient satisfaction, patients’ expectations, quality of care

## Abstract

**Aim:**

To evaluate patients’ satisfaction with the quality of nursing care and examine associated factors.

**Design:**

A cross‐sectional, descriptive survey study.

**Methods:**

The sample was composed of 635 patients discharged from a private hospital. Data were collected using “Patient Satisfaction with Nursing Care Quality Questionnaire” with a total of 19 items, and a questionnaire designed to record socio‐demographic characteristics and medical histories between January 1–May 31, 2015.

**Results:**

Patients were more satisfied with the “Concern and Caring by Nurses” and less satisfied with the “Information You Were Given.” Patients (63.9%) described nursing care offered during hospitalization as excellent. Patients who were 18–35 years old, married, college or university graduates, treated at the surgery and obstetrics–gynaecology units, and patients who stated their health as excellent and hospitalized once or at least five times were more satisfied with the nursing care. According to this study, the nurses needed to show greater amount of interest to the information‐giving process.

## 1 INTRODUCTION

Increasing competition in every field today also affects the healthcare industry. The most important competitive advantage of health service providers is to provide quality health services (Alsaqri, 2016; Reck, 2013; Şişe, 2013). The need for increased quality of healthcare services has been identified via health‐related information and advances in technology, changes in expectations and opinions about health care, an increase in individuals’ involvement in their health care and increased cost and competitiveness in the health sector (Freitas, Silva, Minamisava, Bezerra, & Sousa, 2014).

The quality and adequacy of healthcare services can be measured based on views and satisfaction of patients and their relatives (Merkouris et al., 2013). Patient satisfaction is the most important indicator of quality of care and it considered an outcome of healthcare services (Abdel Maqsood, Oweis, & Hansa, 2012; Akhtari‐Zavare, Abdullah, Syed Hassan, Binti Said, & Kamali, 2010; Mohanan, Kaur, Das, & Bhalla, 2010). Patient satisfaction measurement provided crucial information on performance thus contributing to total quality management (Goh, Ang, Chan, He, & Vehvilainen Julkunen, 2016; Shinde & Kapurkar, 2014). Total quality management includes professional knowledge, competence and application of appropriate technology, the patients’ perception about the type and level of the care they have received (Özsoy et al., 2007; You et al., 2013). In today’s consumer‐oriented healthcare markets, a patient‐centred measure of satisfaction with the quality of nursing care received is a major component of hospital quality management systems (Laschinger, Hall, Pedersen, & Almost, 2005). Patients need their problems diagnosed and treated properly, their function restored and/or symptoms relieved. If the results are unsatisfactory, consumers will change the healthcare facility they applied for treatment and care (Ksykiewicz‐Dorota, Sierpińska, Gorczyca, & Rogala‐Pawelczyk, 2011; Shinde & Kapurkar, 2014). Patients who are more satisfied with their care are more likely to follow medically prescribed regimens and thus contributing to the positive influence on health (Buchanan, Dawkins, & Lindo, 2015; Dzomeku, Atinga, Tulukuu, & Mantey, 2013; Fröjd, Swenne, Rubertsson, Gunningberg, & Wadensten, 2011). More satisfied patients are more liable to recommend the hospital to family and friends (Buchanan et al., 2015; Mohanan et al., 2010). Patients’ opinions are the best source that can tell the providers of what is important, that is why this information can be used in healthcare planning and evaluation (Abdel Maqsood et al., 2012; Alsaqri, 2016; Merkouris et al., 2013; Villarruz‐Sulit, Dans, & Javelosa, 2009). All these changes and developments in the healthcare field require restructuring of all healthcare services, including nursing, through questioning the quality of treatment services (Şişe, 2013).

### 1.1 Background

Patient satisfaction is a concrete criterion for evaluation of health care and therefore quality of nursing care (Alhusban & Abualrub, 2009; Shinde & Kapurkar, 2014). It provides crucial information for healthcare managers by providing important resources for processes such as those involved in measuring patients’ expectations and satisfaction with nursing care quality, improving nursing service quality through identification of areas of failure and planning and implementing necessary training (Abdel Maqsood et al., 2012; Gadalean & Cheptea, 2011; Geçkil, Dündar, & Şahin, 2008). Evaluation of health care involves defining the objectives of care, monitoring healthcare inputs, measuring the extent to which the expected outcomes have been achieved and assessing the extent of any unintended or harmful consequences of the intervention (Alsaqri, 2016; Sitzia & Wood, 1997; Tang, Soong, & Lim, 2013).

Nursing care is one of the major components of healthcare services (Buchanan et al., 2015; Merkouris et al, 2013; Mohanan et al., 2010; Sitzia & Wood, 1997). Patients’ satisfaction with nursing care has become an established as the most important predictor of the overall satisfaction with hospital care and an important goal of any healthcare organization (Goh et al., 2016; Laschinger et al., 2005; Mohanan et al., 2010; Reck, 2013). Measuring patients’ satisfaction with nursing care could be effective in improving nursing service quality by facilitating the creation of standards for care while monitoring both results and patients’ perceptions of quality (Akın & Erdoğan, 2007; Senarath & Gunawardena, 2011; Tang et al., 2013). The nurses have a central role in offering emotional and psychological support to patients and their families in all settings, such as supporting the patient through diagnosis and ensuring optimum care given to them. Besides the provision of technical care, nurses must have the qualified professional knowledge, attitudes and skills, providing the informational, emotional and practical supports (Akhtari‐Zavare et al., 2010; Buchanan et al., 2015; Goh et al., 2016).

If healthcare organization managers are able to identify patient expectations, they could accordingly adjust the performance of services that they offer to meet these expectations (Freitas et al, 2014; Fröjd et al., 2011; Milutinovic, Simin, Brkic, & Brkic, 2012). The surveys in health services concerning health satisfaction are carried out to evaluate the patient satisfaction, to learn patient’s expectations, their suggestions and feedbacks, make the quality improvement constantly in all service periods, to search the effects of socio‐demographic and treatment periods on patient satisfaction (Buchanan et al., 2015; Özer & Çakıl, 2007; Sitzia & Wood, 1997). That is why patient satisfaction should be measured constantly using valid, reliable assessment instruments to assess care quality, identify variables that affect care and determine which items should be prioritized and which require alteration in the service based on patients’ responses (Buchanan et al., 2015; Merkouris et al., 2013). A good assessment instrument measuring the factors that determine patient satisfaction should be developed to improve nursing service quality (Freitas et al., 2014; Laschinger et al., 2005). Therefore, the findings of nursing management research should be used as an indicator of the contribution made by nursing to the patient care process and this could aid the advancement of the profession in terms of scientification (Alsaqri, 2016; Freitas et al., 2014; Goh et al., 2016).

### 1.2 Research questions

- What is the satisfaction level of patients about the quality of nursing care?
- Is there any relationship between patients’ satisfaction with the quality of nursing care according to their socio‐demographic characteristics and medical history?

## 2 METHODS

### 2.1 Design

The study used a descriptive, cross‐sectional research design.

### 2.2 Setting and samples

Participants included 635 hospitalized patients receiving internal medicine, surgery and obstetrics and gynaecology services at a private hospital between January 1–May 1, 2015. The sampling criteria were as follows: patients aged 18 years or older, patients who were discharged, hospitalized for at least 2 nights at the time of data collection, able to speak and understand Turkish, not too confused or ill to complete the questionnaires and agreeing to participate in the study.

The response rate of this study is 92.8%. The survey was not administered to all patients who had not planned their discharge (those were decided or wished to be discharged suddenly) or were transferred to another hospital. Incompletely filled out surveys were not included in the study.

### 2.3 Ethical considerations

Prior to data collection, the research protocol was reviewed and approved by the relevant scientific ethics committee (IBU Clinical Research Ethical Committee, Ethical Approval Number: 01.11.2014/25‐168). Permission to conduct the research was also obtained from hospital administrators. Written approval to use the Patient Satisfaction with Nursing Care Quality Questionnaire (PSNCQQ) and translate it into Turkish was obtained from Laschinger, who developed the scale. All patients provided written informed consent.

### 2.4 Measurement

#### 2.4.1 Patient Satisfaction with Nursing Care Quality Questionnaire

The PSNCQQ was designed to measure the extent of anticipated need, assess patient satisfaction following short‐stay hospitalization and determine the influence of socio‐demographic, personal and other factors at a minimum level. The scale was developed using the Patient Judgements of Hospital Quality Questionnaire, which was developed by a multidisciplinary research team at the Hospital Corporation of America (Laschinger et al., 2005; Reck, 2013).

The scale consists of 19 items pertaining to features of a wide range of nursing activities including nurses’ attention, kindness, respect, courtesy, skills, competence and fulfilment of patient needs. As it is short and it can be completed easily, it has very good psychometric properties that can be used by managers in quality improvement activities (Fröjd et al., 2011; Laschinger et al., 2005). Each item consists of a “signpost,” which is a phrase designating its content and a “descriptor,” which is a detailed question. The scale also includes a general perceptions section consisting of four additional questions designed to measure satisfaction with the overall quality of care and treatment received during hospitalization, the overall quality of nursing care, thoughts on overall health and the likelihood that the patient would recommend the hospital to relatives and friends (Laschinger et al., 2005; Milutinovic et al., 2012).

The scale was designed for application by administrators in areas requiring improvement, to provide patient‐oriented outcomes and for the identification of strong and weak aspects of the nursing care process. Items were based on factors identified as important elements of patient satisfaction with nursing care. The PSNCQQ can be incorporated into existing hospital quality monitoring systems to monitor patient satisfaction. In addition, the PSNCQQ can be used as an evidence‐based indicator given its contribution to the patient care process as a result variable, to evaluate changes in departmental and institutional processes. This feedback provides useful information to nurse administrators (Abdel Maqsood et al., 2012; Laschinger et al., 2005).

Participants’ responses are provided using a 5‐point Likert‐type scale. Total possible scores range from 19–95. Lower total scores indicate greater satisfaction with nursing care. The scoring of the scale was: 1 = excellent, 2 = very good, 3 = good, 4 = fair, 5 = poor.

### 2.5 Data collection

Data were collected using the PSNCQQ, which measures health‐related properties considered to affect patient satisfaction and a questionnaire, designed in the light of related literature, to record socio‐demographic characteristics and medical history. A questionnaire consisting of 16 items pertaining to variables affecting patient satisfaction was developed according to these characteristics. Income levels were measured by the patients’ self‐perception of their economic status and lifestyle. It was presented in four options: low, moderate, high and very high. Perceived health was measured by a self‐reported question was graded by six variables prior to their admission as excellent, good, fair, poor, very poor and unsure.

The data were collected by the researcher. The patients completed the questionnaires prior to their discharge from the hospital. The patients who agreed to participate in the study were provided with an explanation about the purpose of the study and they signed informed consent forms. Those who refused to participate reported that they did not have the time or were just not interested in participating. Data were collected by face to face interviews from illiterate patients.

### 2.6 Data analysis

Data were analysed using SPSS software (IBM Corp. Released 2012. IBM SPSS Statistics for Windows, Version 21.0; IBM Corp, Armonk, NY, USA). The analysis included descriptive statistics such as frequencies, means, standard deviations and percentages. The distribution of the data was assessed using the Single Sample Kolmogorov–Smirnov test and as the significance values exceeded 0.05, parametric tests were used in the advanced‐level analysis. About the parametric tests, *t* tests were performed to analyse independent variables with two categories, one‐way ANOVAs were performed to analyse independent variables with more than two categories and Pearson’s correlation coefficients were used to analyse relationships.

### 2.7 Validity and reliability analysis

The PSNCQQ was translated into Turkish and the linguistic, and conceptual equivalence of the items was established. Back translation was performed to ensure language equivalence between the English and Turkish versions of the scale. The original scale was translated into Turkish linguists who were highly competent in both languages. Five bilingual experts consisted of a doctor, two nursing faculty members, a nurse manager and a linguist. Expressions used in the scale were analysed individually and in combination and optimal expressions were selected by forming a pool of 19 items. Back translation from Turkish to English was performed by two trained linguists (English teachers) with knowledge and experience in both languages. The back‐translated and original versions of the PSNCQQ were compared and found to be highly similar in meaning and reorganized based on the characteristics of the country. After then, the expert met and reviewed to determine the scope of the validity of the scale. Eight experts (nursing academicians specialized in medical nursing, surgical nursing and nursing administration) provided opinions about meaning and content sufficiency. A pilot study was then conducted from 1–31 December 2014 to determine whether there were any unclear questions in the scale. The data from the pilot study were then excluded from the final data analysis. According to the results of the pilot study, small changes were then made to the expressions in some scale items to increase their understandability.

In this study, the coefficients for correlations between average PSNCQQ item scores ranged from 0.80–0.89, which demonstrated an appropriate level of reliability. Cronbach’s *α* for the PSNCQQ, calculated to determine internal consistency and uniformity, was 0.98, which was very high. In Laschinger et al.’s (2005) study, the coefficients for the correlations between PSNCQQ items ranged from 0.61–0.89 and were described as high and Cronbach *α* was 0.97, which was described as excellent. Therefore, the results obtained in the current study were similar to those reported by Laschinger et al. (2005). In view of this, the Turkish version of the PSNCQQ could be considered to possess excellent psychometric properties, which were similar to those reported for the original scale.

## 3 RESULTS

### 3.1 Socio‐demographic characteristics and medical history

The mean age of the sample age was 47.94 (*SD* 19.66) years and 37.6% were aged between 18–35 years. The most of participants were women (77.3%), married (74.5%) and college or university graduates (33.2%), at moderate‐income level (52.1%) and housewives (31.3%). Of the patients, 2.2% were illiterate. More than half of the sample (61.6%) were admitted to the service directly from the patient admission department and had been hospitalized once in the preceding 2 years (66.6%). The average duration of the current hospitalization was (4.38 *SD* 5.75) days (Table 1).

**Table 1:** Patient characteristics (*N* = 635)

Variables	*N*	%
Age (years)		
18–35	239	37.6
36–55	180	28.3
56 and more	216	34.0
Gender		
Female	491	77.3
Male	144	22.7
Marital status		
Married	473	74.5
Single	65	10.2
Divorced	22	3.5
Widowed	75	11.8
Education		
Illiterate	14	2.2
Literate	16	2.5
Primary school	114	18.0
Secondary school	54	8.5
High school	200	31.5
College or University	211	33.2
Postgraduate	26	4.1
Perceived income level		
Very high	19	3.0
High	268	42.2
Moderate	331	52.1
Low	17	2.7
Occupation		
Worker (blue collars)	47	7.4
Civil servant	47	7.4
Retired	109	17.2
Self‐employment	73	11.5
Housewife	199	31.3
Student	17	2.7
Others	143	22.5

### 3.2 PSNCQQ** scores**

Analysis of PSNCQQ scores revealed that the item for which satisfaction levels were highest (1.38 *SD* 0.66) was the “Concern and Caring by Nurses: Courtesy and respect you were given; friendliness and kindness” item. The item for which satisfaction levels were lowest (1.74 *SD* 0.86) was the “Information You Were Given: How clear and complete the nurses’ explanations were about tests, treatments and what to expect” item. Overall, patients’ PSNCQQ scores ranged between 1–4.05, with an average score of 1.61 (*SD* 0.65). This indicated that the level of satisfaction with nursing care was high (Table 2).

**Table 2:** Distribution of Patient Satisfaction with Nursing Care Quality Questionnaire (PSNCQQ) Scores (*N* = 635)

Items	M	*SD*	Min	Max
1. Information You Were Given: How clear and complete the nurses’ explanations were about tests, treatments and what to expect	1.74	0.86	1	5
2. Instructions: How well nurses explained how to prepare for tests and operations	1.72	0.84	1	5
3. Ease of Getting Information: Willingness of nurses to answer your questions	1.57	0.76	1	5
4. Information Given by Nurses: How well nurses communicated with patients, families, and doctors	1.59	0.74	1	5
5. Informing Family or Friends: How well the nurses kept them informed about your condition and needs	1.72	0.82	1	5
6. Involving Family or Friends in Your Care: How much they were allowed to help in your care	1.71	0.77	1	5
7. Concern and Caring by Nurses: Courtesy and respect you were given; friendliness and kindness	1.38	0.66	1	5
8. Attention of Nurses to Your Condition: How often nurses checked on you and how well they kept track of how you were doing	1.51	0.72	1	4
9. Recognition of Your Opinions: How much nurses ask you what you think is important and give you choices	1.74	0.85	1	5
10. Consideration of Your Needs: Willingness of the nurses to be flexible in meeting your needs	1.57	0.74	1	5
11. The Daily Routine of the Nurses: How well they adjusted their schedules to your needs	1.65	0.77	1	4
12. Helpfulness: Ability of the nurses to make you comfortable and reassure you	1.49	0.70	1	4
13. Nursing Staff Response to Your Calls: How quick they were to help	1.48	0.68	1	4
14. Skill and Competence of Nurses: How well things were done, like giving medicine and handling IVs	1.58	0.79	1	5
15. Coordination of Care: The teamwork between nurses and other hospital staff who took care of you	1.58	0.72	1	5
16. Restful Atmosphere Provided by Nurses: Amount of peace and quiet	1.60	0.81	1	5
17. Privacy: Provisions for your privacy by nurses	1.53	0.73	1	5
18. Discharge Instructions: How clearly and completely the nurses told you what to do and what to expect when you left the hospital	1.63	0.77	1	5
19. Coordination of Care After Discharge: Nurses’ efforts to provide for your needs after you left the hospital.	1.7	0.81	1	5
Average PSNCQQ Score	1.61	0.65	1	4.05

Analysis of PSNCQQ scores for perception‐related items showed that 61.4% and 63.9% of participants’ responses for the “Quality of the care and service provided during your stay at the hospital” and “Quality of the nursing care provided during your stay at the hospital” items, respectively, were “excellent.” In addition, 87.9% of patients stated that they would recommend the hospital to their family and friends.

### 3.3 Comparison of PSNCQQ scores according to patients’ socio‐demographic characteristics

The mean PSNCQQ score of patients at the age of 56 years or older was significantly higher (1.75 *SD* 0.68) in relation to those observed for patients aged 18–35 years (1.50 *SD* 0.61) and aged 36–55 years (1.56 *SD* 0.62; *p* < 0.001). No statistically significant differences were found in the PSNCQQ scores of patients by gender or occupation (*p* > 0.05).

The widowed patients’ mean PSNCQQ score was found to be statistically higher (1.81 *SD* 0.75) than that of the married patients’ (1.57 *SD* 0.62), and the difference was significant (*p* < 0.05). The patients’ PSNCQQ scores varied significantly by education level and income (*p* < 0.001). The literate patients and patients with moderate incomes scores were higher (2.02 *SD* 0.65, 1.71 *SD* 0.68, respectively) than those of the patients who had completed college or university and patients with high incomes (1.52 *SD* 0.60, 1.48 *SD* 0.56, respectively; Table 3).

**Table 3:** Comparison of Patient Satisfaction with Nursing Care Quality Questionnaire scores based on patients’ socio‐demographic characteristics (*N* = 635)

	*N*	*M* ± *SD*	Statistical evaluation	
			*t*/*F*	*p*
Age (years)				
18–35	239	1.50 ± 0.61	*F*: 9.506***	<0.001
36–55	180	1.56 ± 0.62		
56 and more	216	1.75 ± 0.68		
Gender				
Female	491	1.59 ± 0.65	*t*: 0.102	0.317
Male	144	1.65 ± 0.63		
Marital status				
Married	473	1.57 ± 0.62	*F*: 3.234*	0.022
Single	65	1.64 ± 0.73		
Divorced	22	1.57 ± 0.55		
Widowed	75	1.81 ± 0.75		
Educational background				
Illiterate	14	2.00 ± 0.77	*F*: 3.042***	0.006
Literate	16	2.02 ± 0.65		
Primary school	114	1.65 ± 0.65		
Secondary school	54	1.71 ± 0.77		
High school	200	1.57 ± 0.61		
College or University	211	1.52 ± 0.60		
Postgraduate	26	1.64 ± 0.75		
Income level				
Very high	19	1.38 ± 0.67	*F*: 7.198***	<0.001
High	268	1.48 ± 0.56		
Moderate	331	1.71 ± 0.68		
Low	17	1.66 ± 0.72		
Occupation				
Worker (blue collars)	47	1.48 ± 0.49	*F*: 1.813	0.094
Civil servant	47	1.41 ± 0.54		
Retired	109	1.70 ± 0.68		
Self‐employed	73	1.61 ± 0.62		
Housewife	199	1.64 ± 0.66		
Student	17	1.75 ± 0.79		
Others	143	1.56 ± 0.65		

Notes

*F*: one‐way ANOVA; *t*: *t* test.

^*^ *p* < 0.05 ^**^ *p* < 0.01 ^***^ *p* < 0.001

### 3.4 Comparison of PSNCQQ scores according to patients’ medical histories

The mean PSNCQQ score of the patients hospitalized in the internal medicine unit was higher (1.95 *SD* 0.75) than those of the patients in the surgery and the obstetrics and gynaecology units (1.51 *SD* 0.57, 1.46 *SD* 0.55, respectively), and the differences were significant (*p* < 0.001). Score for patients admitted to the service from the emergency department was significantly higher (1.90 *SD* 0.69) relation to those observed for patients admitted from the patient admission department and through other means (1.54 *SD* 0.62, 1.58 *SD* 0.66, respectively; *p* < 0.001).

The mean score of the patients who had been hospitalized twice in the preceding 2 years was higher (1.70 *SD* 0.71) than those of the patients who had only been hospitalized once and more than five times (1.55 *SD* 0.61, 1.35 *SD* 0.57, respectively), and the differences were significant (*p* < 0.001). The mean scores of the patients who perceived their health status very poor, poor, fair and good were higher (1.99 *SD* 0.69, 1.67 *SD* 0.64, 1.65 *SD* 0.66 and 1.60 *SD* 0.64, respectively) than in excellent health (1.31 *SD* 0.55), and the differences were significant (*p* < 0.001). Also, the results showed that the duration of hospitalization was significantly associated with PSNCQQ scores; however, the correlation was weak (*r*_p_: 0.195, *p* < 0.01; Table 4).

**Table 4:** Comparison of Patient Satisfaction with Nursing Care Quality Questionnaire scores according to patients’ medical histories (*N* = 635)

	*N*	*M* ± *SD*	Statistical evaluation	
			*t*/*F*	*p*
Unit in which the patient is hospitalized				
Surgery	212	1.51 ± 0.57	*F*: 36.35***	<0.001
Obstetrics and gynaecology	261	1.46 ± 0.55		
Internal medicine	162	1.95 ± 0.75		
Manner of admission to ward				
From emergency department	70	1.90 ± 0.70	*F*: 4.80***	0.001
Directly from patient admissions department	391	1.54 ± 0.62		
After daily operations and tests	52	1.67 ± 0.68		
Transfer from another healthcare facility	28	1.69 ± 0.56		
Other	94	1.58 ± 0.66		
Status of hospitalization in the last 2 years				
Once	423	1.55 ± 0.61	*F*: 3.90***	0.004
Twice	143	1.70 ± 0.71		
Three times	42	1.81 ± 0.66		
Four times	9	1.94 ± 0.67		
Five times or more	18	1.35 ± 0.57		
Perceived health				
Excellent	68	1.31 ± 0.55	*F*: 4.91***	<0.001
Good	314	1.60 ± 0.64		
Fair	160	1.65 ± 0.66		
Poor	63	1.67 ± 0.64		
Very poor	22	1.99 ± 0.69		
Unsure	8	1.59 ± 0.71		

Note

*F*: one‐way ANOVA; *t*: *t* test.

^*^ *p* < 0.05 ^**^ *p* < 0.01 ^***^ *p* < 0.001

## 4 DISCUSSION

The results of this study revealed similarities and differences with the existing national and international literatures. This issue has been discussed as follow.

### 4.1 Discussion of finding about the PSNCQQ scores

Measures of patient satisfaction can assess communication in the consultation such as information transfer, patient involvement in decisions and reassurance (Goh et al., 2016; Shinde & Kapurkar, 2014). Effective and continuous interaction and communication are critical determinants in patients' satisfaction, hospital stay and recovery (Koç, Sağlam, & Şenol, 2011; Mohanan et al., 2010; Negarandeh, Bahabadi, & Mamaghani, 2014; Villarruz‐Sulit et al., 2009). Health professionals’ communication skills play a pivotal role in ensuring that patients feel valued and cared for. The allocation of sufficient time for talking and listening to patients and providing information is a prerequisite for patient satisfaction, as it ensures that patients are less stressed and more engaged and well adjusted (Koç et al., 2011). There is evidence that the health professionals are perceived as communicating well when the patient feels he/she shows individualized interest, understanding and reassurance (Sitzia & Wood, 1997). A study (Abdel Maqsood et al., 2012) indicated that patients were more satisfied with having respectful communication whereas they were less satisfied with the professional information provided by the nurses about their disease, health status, investigations and prognosis of their condition. In a meta‐analysis conducted by Özsoy et al. (2007), patients expected favour, attention, understanding, kindness and helpfulness from individuals providing care services. In our study, the highest level of satisfaction, represented by PSNCQQ scores, was reported for the “Concern and Caring by Nurses” item. The results indicate that the nurses’ communication style is to treat patients respectfully and be friendly towards them. However, the nurses were less interested in explanations about their interventions and communication with patients that did not meet their expectations.

Information provision and education are important factors affecting patient satisfaction (Abdel Maqsood et al., 2012; Koç et al., 2011; Villarruz‐Sulit et al., 2009). Nurses and other healthcare professionals play a key role in providing support and information. Nurses care for the patients on a 24‐hr basis and should be empowered to provide requisite information and instructions to the patients (Alhusban & Abualrub, 2009; Shinde & Kapurkar, 2014). Patient education has been linked with positive clinical outcomes such as improved adherence to a therapeutic regime, reduced anxiety and enhanced ability to cope with symptoms (Sitzia & Wood, 1997). It is known that receipt of adequate information affects patients’ confidence and satisfaction and this is the most important factor in encouraging patients to participate in their own health care. In addition, providing patients and their families with information about patients’ conditions is important in helping them overcome fear of the unknown (Dzomeku et al., 2013; Koç et al., 2011; Milutinovic et al., 2012). Several studies have reported inadequacies in information provision. For example, Dzomeku et al. (2013) found that the type and amount of information provided by nurses about patients’ conditions constituted one of the main causes of dissatisfaction. In a meta‐analysis conducted by Özsoy et al. (2007), the patients’ most important expectation concerning care quality was that they should be informed about medication and treatment. Patients reported that information played an important role in their satisfaction and they emphasized that information provided by nurses should be clear and concise. Therefore, it is crucial for nurses to realize that information provision and education are nursing responsibilities and that they should collaborate with other healthcare staff to provide complete and relevant information to patients. Abdel Maqsood et al. (2012) indicated that the patients had low levels of satisfaction with information and instructions given by nurses and nurses had the perception that “information giving” was the role of the physicians and the nurses may be fearful to provide information because of the power hierarchy between the nurses and the physicians. In this study, the lowest level of satisfaction, represented by PSNCQQ scores, was reported for the “Information You Were Given” explanations were about tests, treatments and what to expect” item. This result indicates that the explanations and information provided by nurses at the hospital were unsatisfactory in the nursing care.

### 4.2 Discussion of finding about to the PSNCQQ scores according to patients’ socio‐demographic characteristics

Different studies indicated that older patients are generally more satisfied (Dzomeku et al., 2013; Fröjd et al., 2011; Milutinovic et al., 2012). Sitzia and Wood (1997) stated in their review study that older people tend to be more satisfied with health care than younger people are. Similarly, according to Shinde and Kapurkar (2014) older respondents were more satisfied, probably because they were more social and accepting than younger or they had more respect and care for providers. On the contrary, we found that patients aged 56 years or older were less satisfied than other age groups. This can be related to the fact that the nurses did not pay more attention to elderly patients. Another possible reasons can be that levels of satisfaction could differ according to cultural values or the patients did not held positive attitudes towards events, based on age‐related increases in tolerance and maturity levels.

Similarly, in our study, Sitzia and Wood (1997) found that patient gender did not affect satisfaction values and a conclusion reached also in the reports that significantly more men than women. In other studies (Alsaqri, 2016; Arslan & Kelleci, 2011), no relationships were found between gender and patient satisfaction levels. However, while some of these studies (Akın & Erdoğan, 2007; Alhusban & Abualrub, 2009) reported that women’s levels of satisfaction with care were higher relative to those observed in men, others (Koç et al., 2011; Milutinovic et al., 2012; Shinde & Kapurkar, 2014) showed higher satisfaction levels in men relative to those observed in women. In addition, in a study conducted by Dzomeku et al. (2013), 38% and 30% of hospitalized men and women, respectively, were completely satisfied with their nursing care. While the reason for these differences can involve cultural characteristics, they can also occur because, relative to men, women pay more attention to hygiene and care and are more anxious.

In this study, college or university graduates were more satisfied relative to those who were literate patients. However, in some other studies (Dzomeku et al., 2013; Geçkil et al., 2008; Milutinovic et al., 2012; Özsoy et al., 2007), literate individuals and primary school graduates reported greater satisfaction with nursing services relative to that reported by college or university graduates. In addition, Sitzia and Wood (1997) indicated that greater satisfaction was associated with lower levels of education. Patients with lower levels of education being most satisfied, similarly, showed that higher educational attainment was strongly associated with dissatisfaction. Some studies (Akın & Erdoğan, 2007; Shinde & Kapurkar, 2014) revealed that the level of education was not associated with patient satisfaction. These study findings indicated that patients expect more from nursing and care as their education levels increase. This can occur because patients with high educational levels possess more information about treatment alternatives and expect higher care standards and therefore are more critical in this regard.

Patients with high incomes tend to anticipate an improvement in their symptoms and expect to receive care from highly qualified staff and they become dissatisfied if they receive care that does not meet their expectations. Patients with low incomes had low health, get lower health care, had less continuous relation with doctors and have difficulties in getting appointments (Shinde & Kapurkar, 2014). Some studies (Akhtari‐Zavare et al., 2010; Arslan & Kelleci, 2011; Özsoy et al., 2007) reported that satisfaction with nursing care did not differ significantly according to income. In our study, patients with high incomes were more satisfied relative to those with moderate incomes. We can say that these patients received care in the direction of their expectations.

### 4.3 Discussion of finding about to the PSNCQQ scores according to patients’ medical histories

Patients who were hospitalized in surgery and obstetrics and gynaecology units were more satisfied relative to those hospitalized in the internal medicine unit. Shinde and Kapurkar (2014) found that the gynaecological ward had a significantly higher percentage of patients’ satisfaction with nursing care than the surgical wards. In a study conducted by Alhusban and Abualrub (2009), the patients hospitalized in an obstetrics and gynaecology unit reported higher satisfaction levels relative to those hospitalized in internal medicine and surgical units, while in a study conducted by Geçkil et al. (2008), patients hospitalized in surgical units reported higher satisfaction levels relative to those hospitalized in obstetrics and internal medicine units. In the other studies (Akın & Erdoğan, 2007; Koç et al., 2011; Tang et al., 2013), satisfaction scores for patients treated in internal medicine units were higher relative to those treated in surgery units. The difference in dissatisfaction between the types of units occurred because of problems experienced during surgical procedures in conjunction with medical diagnoses and socio‐demographic characteristics. All of these differences can be the levels of physical and psychological dependency on the hospital.

The results of the present study revealed that the patients who hospitalized once or at least five times in the preceding 2 years were more satisfied relative to those hospitalized twice in the preceding 2 years. Alsaqri (2016) showed that there was a statistically significant difference between previous admissions and patient satisfaction levels. The same study demonstrated patients with a history of admission to hospital during the last 2 years found nurses more caring. It seems that more lengths of stay in hospital increase patients’ opportunities for receiving more nurses’ care and observing their caring behaviours. Similarly, in these studies (Koç et al., 2011; Milutinovic et al., 2012) satisfaction levels reported by patients who had been hospitalized previously were higher relative to those who had not. In contrast, in a study conducted by Arslan and Kelleci (2011) satisfaction levels reported by patients with previous hospital experience were lower relative to those without previous hospital experience. The result of another study (Akın & Erdoğan, 2007) found no statistical relationship between satisfaction with nursing care and the numbers of hospitalization. According to these results, we can say that patients’ expectations can vary according to previous experience in similar situations and as the number of admissions increase, they can compare their care with that received previously. Also, the positivity or negativity of patients’ previous experience can be reflected in their approach to current care.

A study (Alsaqri, 2016) indicated that people who perceived themselves as being healthy were more likely to be satisfied with access to care. According to Alsaqri (2016), patients who perceived themselves to be in excellent or good health are more likely to be satisfied with their health care. Also, it is indicated in the same study that, a person’s health prior to arrival at hospital, whether through accident, a chronic condition or a voluntary procedure may affect the patients’ expectations about the care. In addition, Laschinger et al. (2005) reported that patients with good health status postdischarge report greater satisfaction than those with poor health status. Similarly, in our study, patients with very poor, poor, fair or good health were less satisfied relative to those of patients with excellent health. This may be due to the fact that healthier people do not need as much medical care and they interact with healthcare providers less frequently. They have less opportunity to experience problems with access to health care and therefore may express more satisfaction with access.

### 4.4 Study limitations

The sample was restricted to patients from the general surgery, obstetrics and internal medical units. In addition, the study was conducted in a single private hospital in Turkey. Therefore, the results cannot be generalized to all hospitals. Future studies should include more than one hospital in both the private and public sectors and the nursing care provided in private and public hospitals should be compared.

Test–retest reliability analysis should have been performed to strengthen the results of the study. Therefore, patients should be surveyed for a second time in 2 weeks of discharge and the results should be tracked and addressed in future studies. Although methodological problems, such as poor return rates and an inability to collect tracking data for all participants occurred in the study, the results could be considered useful because of the stability criterion for patient satisfaction surveys.

## 5 CONCLUSION

The results revealed that nurses should inform patients about each application and procedure and provide necessary explanations about illness, diagnosis and treatment to ensure patient satisfaction and the provision of high‐quality nursing care. The results also showed that nurses should provide care in a framework of respect, favour and courtesy towards patients by emphasizing the importance of communication. Besides these, the patients were highly satisfied with overall quality of hospital care, nursing care and reported that they would recommend this hospital to their families and friends.

Nurse managers could contribute to the quality service provision by evaluating the patient satisfaction with nursing care for the development and improvement of nursing care based on patients’ expectations. Data obtained from this evaluation should be considered in determining training requirements for nurses and in‐service training programs should be organized to develop nurses’ knowledge and skills in care planning. The PSNCQQ is considered useful for nurse administrators in improving nursing care. The scale could allow managers to determine the attitudes of individuals with whom they work and those whom they manage and exert some degree of control over employees’ behaviour.

## CONFLICT OF INTEREST

The authors declare that there was no conflict of interests.

## AUTHOR CONTRIBUTIONS

AK, ZD: Study design. AK, ZD: Data collection and analysis. AK, ZD: Manuscript preparation.
